# Seed Oil of *Brucea javanica* Induces Apoptotic Death of Acute Myeloid Leukemia Cells via Both the Death Receptors and the Mitochondrial-Related Pathways

**DOI:** 10.1155/2011/965016

**Published:** 2011-07-02

**Authors:** Hong Zhang, Jing Yu Yang, Fan Zhou, Li Hui Wang, Wen Zhang, Sha Sha, Chun Fu Wu

**Affiliations:** ^1^Department of Pharmacology, Key Laboratory of New Drug Screening of Liaoning Province, Shenyang Pharmaceutical University, Shenyang 110016, China; ^2^Department of Hematology, Shenyang Military General Hospital, Shenyang 110016, China

## Abstract

Seed oil of *Brucea javanica* (BJO) is extracted from the seeds of herb medicine *Brucea javanica* (L.), and its emulsion formulation (BJOE) has been used clinically to treat carcinomas for many years in China. The antileukemia potential of BJO was investigated in human acute myeloid leukemia cell lines (AML) U937 and HL-60 *in vitro* and in a mouse U937 xenograft tumor model. BJO induced AML cell apoptosis through activation of caspase-8 and modulation of apoptosis-related proteins. Meanwhile, the inhibition of survivin and XIAP increased the cytotoxicity of BJO. Consistent with these findings, BJO also increased subG_1_ phase cells and cause PARP cleavage in AML patients' leukemia cells. In contrast, only weak cytotoxicity of BJO was found in peripheral blood lymphocytes (PBLs) of healthy volunteers. Moreover, oleic acid and linoleic acid were found to be the active components of BJO. Our study provided strong evidence for the first time that BJO induced apoptosis of both cultured and primary AML cells. Furthermore, intravenous injection of BJO significantly inhibited U937 tumor growth in the xenograft mouse model. These results suggest that BJO may have a therapeutic role in the treatment of human leukemia.

## 1. Introduction

Seed oil of* Brucea javanica* (BJO) is extracted from the seeds of *Brucea javanica *(L.), a traditional herbal medicine, by using circulation extraction method in petroleum ether. Its emulsion formulation, *Brucea javanica* oil emulsion (BJOE), has been used clinically to treat carcinomas for many years in China; its clinical indications include lung cancer, prostate cancer, and gastrointestinal cancer [1–3]. 

Previous studies have found that BJO can induce cytotoxic effects through apoptosis induction. It has been reported that BJO induced apoptosis at low concentrations and induced necrosis at higher concentrations [4]. BJO induces apoptosis via downregulating expression of p53, Bcl-2, c-myc in hepatocellular and bladder cells [2, 4] and upregulating expression of Fas in leukemia cells [5]. Moreover, BJO can arrest the cell cycle in G0/G1 phase [6, 7] to inhibit cell growth. It seems that BJO is a potent apoptotic agent to cancer cells. However, the active components of BJO are still not clear. Also, whether BJO can be used to treat acute myeloid leukemia (AML) and its underlying mechanisms are still unknown. 

Although conventional chemotherapy of AML with either cytarabine or daunorubicin given as a single agent induces complete remission in ~30% to 40% of patients and combination treatment with both agents induced complete remission in more than 50% of patients [8]; however, only 20% to 30% of patients enjoy long-term disease-free survival [9]. Thus, there is a need for new agents for the treatment of AML.

 It has been estimated that approximately 65% of clinically used anticancer agents have a root of natural products [10]. Both homoharringtonine and etoposide are plant original medicines which have significant antileukemia effect [11–13] in clinical studies in [14]. However, clinical studies of these two products showed that myelosuppression, cardiovascular, and gastrointestinal events were usually accompanied with their anticancer effects [14]. While, in the clinical studies of chemotherapy combined with the emulsion of BJO, it has been reported that the toxicity of combination group is much less than the chemotherapy group [15, 16]. Therefore, to examine the potential of BJO for clinical applications in the treatment of AML and identify the active components of BJO would be very significant. 

In this study, our results show that BJO induces apoptosis through both mitochondrial pathway and death receptor pathway. Its apoptotic effect on AML cells was much more potent than on normal PBLs from healthy volunteers. BJO can also inhibit tumor growth in mouse U937 xenograft tumor model and induce apoptosis in AML patients' leukemia cells. These combined observations suggest that BJO has a high potential for clinical application in the treatment of AML. The major active components of BJO were also identified as oleic acid and linoleic acid in our study.

## 2. Materials and Methods

### 2.1. Reagents

*Brucea javanica* oil emulsion (Lot no. 060724) and soybean phospholipids (Lot no. 060724, emulsifying agent) were provided by Shenyang Yaoda Pharmaceutical Co. Ltd. (Shenyang, Liaoning, China). Oleic acid (cell culture tested, Lot no. O1383) and linoleic acid (cell culture tested, Lot no. L1012) were purchased from Sigma-Aldrich (St. Louis, MO).The etoposide emulsion was generously donated by Prof. Xing Tang from the Department of Pharmaceutics, Shenyang Pharmaceutical University. Trizol was from Invitrogen Corporation (USA) and the Revert Aid Mulv RT Kit was from Fermentas Inc. (Hanover, USA). Tag DNA polymerase and primers were from Takara (Tokyo, Japan) while the poly-(ADP-ribose)-polymerase (PARP) antibody was from Boehringer Mannheim (Indianapolis, IN). The antibodies for caspase-3 and Bcl-2 were purchased from BD Biosciences (San Diego, CA), while the antibodies for Bax and *β*-actin were from Santa Cruz Biotechnology (Santa Cruz, CA). The other antibodies were from Cell signaling Technology Inc (UK). Lympholyte-human 1077 (15 min, 280× g) was from Tianjin TBD Biotek Ltd. Co. (Tianjin, China). 

Seven to eight-week-old male Balb/c nude mice were purchased from National Rodent Laboratory Animal Resources (Beijing). All other reagents were purchased from Sigma (St. Louis, MO).

### 2.2. The Preparation of Brucea Javanica Oil Emulsion (BJOE)

The BJO was extracted from the seeds of *Brucea Javanica* by using circulation extraction method in petroleum ether (boiling range: 60–90°C). After extracting, the extraction was heated to evaporate petroleum ether and crude BJO was obtained. The active carbon and crude BJO were mixed together and the mixture was kept 90°C for 30 minutes. When cooling to 80°C, the mixture was filtered through a stainless steel Büchner's funnel with medium speed filter paper and clarifying filter paper. The refined BJO was emulsified by adding soybean phospholipids. (The prescription and preparation methods were provided by Shenyang Yaoda Pharmaceutical Co. Ltd. Shenyang, Liaoning, China)

### 2.3. Cell Culture

Leukemia cell lines U937 and HL-60 cells were cultured in RPMI 1640 supplemented with 100 units/mL penicillin, 100 *μ*g/mL streptomycin, 1 mmol/L L-glutamine, and 10% heat-inactivated fetal bovine serum. Cells were maintained in 75 cm^2^ flasks in a humidified incubator at 37°C with 5% CO_2_. All these cell lines were generous gifts from Dr. Y. K. Jing, the Mount Sinai School of Medicine, New York, US.

### 2.4. Samples from Patients and Healthy Volunteers

Fifteen newly diagnosed AML patients (M5 and M2) and 4 healthy volunteers entered this study between November 2008 and June 2009 at Shenyang Military General Hospital. The diagnoses were based on clinical data (history, symptoms, and physical findings), examination of peripheral blood, and bone marrow according to the French-America-British (FAB) classification and karyotype identification. The main clinical patient data at diagnosis are summarized in Table 1. Blood samples were taken from nonsmoking healthy volunteers who had not been treated with any anticancer drugs for at least 6 months. The study was approved by the Medical Ethical Committee of Shenyang Military General Hospital, and informed consent was obtained from each patient and volunteer.

### 2.5. Isolation of Human Peripheral Blood Lymphocytes

Human peripheral blood lymphocytes were isolated as described previously [17]. In brief, after diluting blood with PBS, lymphocytes were isolated by centrifugation over a density gradient of lympholyte-human 1077 for 15 min at 280 g. The cells were washed with PBS twice then suspended in complete RPMI 1640 with 10% fetal bovine serum. The viability of the isolated lymphocytes was measured by trypan blue exclusion assay and found to be about 99%. 

### 2.6. MTT Assay

Leukemia cells were seeded at a density of 1.5 × 10^5^  on 96-well plates and incubated with emulsifying agent (soybean phospholipids, 1 : 200, v/v), various concentrations of BJO (67.5 *μ*g/mL, 125 *μ*g/mL, 250 *μ*g/mL, 500 *μ*g/mL, 1000 *μ*g/mL), and etoposide (positive control, 10 *μ*mol/L) or oleic acid (37.5 *μ*g/mL, 75 *μ*g/mL, 150 *μ*g/mL) or linoleic acid (56.25 *μ*g/mL, 112.5 *μ*g/mL, 225 *μ*g/mL) for 6 h. Then 10 *μ*L MTT solution (2.5 mg/mL in PBS) was added to each well, and the plates were incubated for an additional 3 h or 4 h at 37°C. After centrifugation (2500 rpm, 10 min), the medium with MTT was aspirated, followed by the addition of 100 *μ*L DMSO. The optical density of each well was measured at 492 nm with a Biotek Synergy TM HT Reader.

### 2.7. Evaluation of Apoptosis

Apoptotic cells were evaluated by morphologic observation, DNA fragmentation assay, and cell cycle examination. For morphologic evaluation, cells were stained with Hochest33258 (10 *μ*g/mL) and AO/EB (10 *μ*g/mL) and observed by fluorescence microscopy. For the DNA fragmentation assay, cells were washed with PBS and harvested by centrifugation. Cells were suspended in 200 *μ*L PBS solution, then 10 *μ*L RNase A (20 mg/mL, Sigma-Aldrich) was added. After the sample had been incubated at 37°C for 45 min, the genomic DNA mini preparation kit with a spin column was used to obtain the genomic DNA. The genomic DNA was separated on a 1.8% agarose gel with 1 *μ*g/mL ethidium bromide. The cell cycle examination was carried out as described elsewhere [18]. In brief, cells were collected and fixed in ice-cold 70% ethanol overnight. Then, the cells were washed with PBS by centrifugation and incubated with 200 *μ*g/mL RNase A for 30 min at 37°C. Before measuring the DNA content by flow cytometry (Becton Dickinson, San Jose, CA), 30 *μ*L propidium iodide solution (1 mg/mL) was added. Data were analyzed using CELLQuest (Becton Dickinson) software.

### 2.8. Examination of Intracellular Reactive Oxygen Species (ROS) Release

Intracellular hydrogen peroxide levels were monitored by flow cytometry after staining with DCFH-DA (6-carboxy-2′,7′-dichlorodihydrofluorescein diacetate) [19]. Briefly, cells in a logarithmic growth phase (1 × 10^5^ cells/mL) were labeled with 5 *μ*mol/L DCFH-DA for 1 h and then treated with various concentrations of BJO. The cells were washed with PBS and then analyzed by flow cytometry (Becton Dickinson, San Jose, CA) with excitation and emission wavelengths of 488 and 525 nm, respectively. Cells stimulated with 100 *μ*m H_2_O_2_ for 1 h were used as a positive control.

### 2.9. Mitochondrial Membrane Potential Assay

The mitochondrial membrane was monitored using rhodamine 123 (Rh123) fluorescent dye (Ex/Em = 507/529) which is selectively taken up by mitochondria in a manner proportional to the mitochondrial membrane potential [20]. In brief, cells treated with various concentrations of BJO were collected and washed with PBS then incubated with 0.5 *μ*g/mL Rh123 in the dark for 30 min at 37°C. The cells were washed with PBS then analyzed by flow cytometry (Becton Dickinson, San Jose, CA).

### 2.10. Reverse Transcription-Polymerase Chain Reaction (RT-PCR)

Total RNA was extracted with Trizol according to the manufacturer's protocol. Total RNA (1 *μ*g) was reverse transcribed to cDNA using a reverse transcription system following the manufacturer's instruction manual. The cDNA was then amplified by PCR with specific primers of Bax sense; 5′-ACC AAG AAG CTG AGC AGT GTC-3′; anti-sense 5′-TGT CCA GCC CAT GAT GGT TC-3′, 258 bp [21], Bcl-2 sense; 5′-CGA CGA CTT CTC CCG CCG CTA CC-3′; anti-sense 5′-CCG CAT GCT GGG GCC GTA CAG TTC C-3′, 318 bp [22], PCNA sense; 5′-AAA CCA GCT AGA CTT TCC TC-3′; anti-sense 5′-TCA CGC CCA TGG CCA GGT TG-3′, 274 bp [23], pro-caspase3 sense 5′-TAA ATC CCA CTG CCA CGG TCG-3′; anti-sense 5′-TGG AAC AAA TGG ACC TGT TGA CC-3′, 453 bp; survivin sense 5′TGC CTG GCA GCC CTT TCT CA 3′; anti-sense 5′ GAT GGC ACG GCG CAC TTT CT 3′, 383 bp and GAPDH sense 5′-TGA TGA CAT CAA GAA GGT GGT GAA G-3′; anti-sense 5′-TCC TTG AGG CCA TGT GGG CCAT-3′, 240 bp [24]. The annealing temperatures were 61°C, 60°C, 55°C, 55°C, and 55°C, respectively. The PCR products were separated on 1.2% agarose gels and visualized with 0.5 *μ*g/mL ethidium bromide.

### 2.11. Western Blotting Analysis

Protein extracts (45 *μ*g) were prepared with radioimmunoprecipitation assay lysis buffer (50 mmol/L Tris-HCL, 150 mM/L NaCL, 0.1% SDS, 1% NP-40, 0.5% sodium deoxycholate, 1 mmol/L phenylmethylsulfonyl fluoride, 100 *μ*mol/L leupeptin, and 2 *μ*g/L aprotinin) and were subjected to electrophoresis on 8% or 12% SDS-polyacrylamide gels and transferred to 0.45 *μ*m nitrocellulose membranes. The membranes were stained with 0.2% Ponceau S red to check equal protein loading and transfer. After blocking with 5% skimmed milk, the membranes were incubated with antibodies to PARP, Bcl-2, Bax, Bid, procaspase-3, caspase-8, c-FLIP_(L/S)_(Cellular FLICE inhibitory protein), XIAP, DR4 (Death Receptor 4), DR5 (Death Receptor 5), MCL-1, or *β*-actin overnight at 4°C. The membranes were incubated with horseradish peroxidase-conjugated secondary antibody, and the immunocomplexes were visualized by ECL western blotting detection reagents. The protein concentrations in the lysate were determined using the BCA protein assay kit.

### 2.12. *In Vivo* Antileukemia Effects on Xenograft Transplantation

To determine the *in vivo* antitumor activity of BJO, viable U937 cells (3 × 10^7^/200 *μ*L PBS per mouse), as confirmed by trypan blue staining, were subcutaneously injected into the right flank of 7- to 8-week-old male Balb/c nude mice. When the average s.c. tumor volume reached 100 mm^3^, the mice were randomly divided into five treatment groups, including control (saline only), etoposide injection (2.5 mg/kg, via tail vein, daily), and BJO (5.5 mg/kg, 16.5 mg/kg, 49.5 mg/kg, via tail vein, daily). Tumor size was measured once every two days with a caliper (calculated volume = shortest diameter^2^ × longest diameter/2). Body weight, diet consumption, and tumor size were recorded once every two days. After 10 days, the mice were sacrificed and the tumors were excised and weighed. One part of tumor tissue was fixed in formalin, and the remaining part was stored at −80°C until further analysis. Blood was collected in heparinized tubes by cardiac puncture.

### 2.13. Measurement of Components in the Emulsion of BJO by Gas Chromatography-Mass Spectrometry

The total components of the emulsion of BJO were determined by gas chromatography-mass spectrometry (GC-MS), and oleic acid was used as a reference. Briefly, sodium chloride and 5 drops of hydrochloric acid were added to BJOE which was kept in a 65°C water bath until oil-water separation. After the solution cooled to room temperature, BJOE was extracted with petroleum ether, and then acetone. Methanolic sodium hydroxide solution (0.5 mol/L) was added to the test solution in the 65°C water bath for saponification until all the oil droplets dissolved. After cooling to room temperature, a 13% methanolic solution of boron trichloride was added to the solution in the 65°C water bath and it was allowed to stand for 15 min. Then, 2 mL N-hexane was added to the test solution in an ice-water bath, and shaken for 2 min. A saturated sodium chloride solution was added until the N-hexane layer rose to the bottle neck and then the N-hexane solution was removed from the bottle. Dried sodium sulfate was added to the N-hexane solution to obtain the test solution. The test solution was then transferred to a Suplecowax-10 fused-silica capillary column (30 m × 250 *μ*m × 0.25 *μ*m), using polyethylene −20 M as a loading agent. The sample-input temperature was 250°C; the detector temperature was 250°C; the column temperature was 220°C; the nitrogen flow rate was 0.5 ml/min and the flow ratio was 20 : 1.

### 2.14. Data Analysis

Results were expressed as the mean ± SE of three experiments. One-way ANOVA followed by Dunnett's *t*-test was used for statistical analysis (SPSS 13.0 software, SPSS, USA).

## 3. Results

### 3.1. BJO-Induced Apoptosis in Acute Myeloid Leukemia Cell Lines

**Figure 1:**
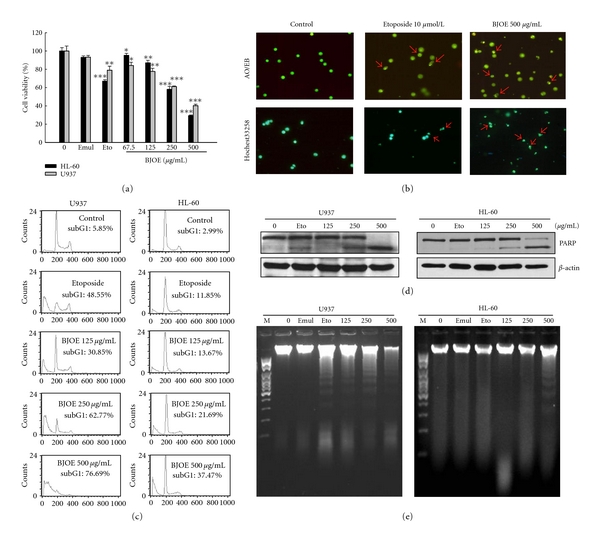
*Cytotoxic effects of BJO on different tumor cell lines.* HL-60 and U937 cells were treated with emulsifier (negative control), etoposide (10 *μ*mol/L, positive control), or various concentrations of BJO as indicated for 6 hours. (a) Cell viabilities were determined by MTT assay. ***P < .001 (b) U937 cell morphology analysis following treatment with BJO. AO/EB and Hochest3325 were used to stain cells and the cellular fluorescent changes were observed using fluorescence microscope. Results were obtained from three separate experiments. (c) Apoptotic cells were evaluated by cell cycle analysis. Percentage of cells in the subG1 phase was measured using flow cytometry as described in Materials and Method. (d) Western blotting analysis of PARP. *β*-actin is shown as a loading control. (e) DNA fragmentation analysis. The level of DNA fragmentation was determined as described in Materials and Method.

In this study, the effect of BJO on the viability of HL-60 and U937 cells was examined by MTT assay. Cells were treated with or without BJO at different concentrations (67.5, 125, 250, and 500 *μ*g/mL). As shown in Figure 1(a), after treatment for 6 h, BJO significantly reduced the cell viability in a concentration-dependent manner with IC_50_ values of 312.7 and 265.4 *μ*g/mL for HL-60 and U937 cells, respectively. Soybean phospholipids (the emulsifier of BJOE) did not affect the cell viability in either of those cell lines. 

The abnormalities of cell morphology were examined using fluorescence microscopy. Cells treated with BJO at different concentrations demonstrated morphology characteristic of apoptosis, such as chromatin condensation and formation of apoptotic bodies (Figure 1(b)). To further investigate the mode of cell death induced by BJO, U937 and HL-60 cells were stained with propidium iodide after BJO treatment. It was shown that BJO produced concentration-dependent significant increases in the proportions of apoptotic cells without G0/G1 or G2/S phase arrest (Figure 1(c)). Moreover, it was found that PARP, a substrate of caspase-3, was also cleaved by BJO (Figure 1(d)). BJO could also induce marked DNA fragmentation in U937 and HL-60 cells (Figure 1(e)), while it had no effect on mRNA level of proliferating cell nuclear antigen (PCNA), an essential component of the replication mechanism (data not shown). All these results indicated that BJO induced apoptotic death of U937 and HL-60 cells.

### 3.2. The Reduction in Mitochondrial Membrane Potential (MMP) Caused by Reactive Oxygen Species (ROS) Production Is Not the Only Mediator of BJO-Induced Apoptosis

**Figure 2:**
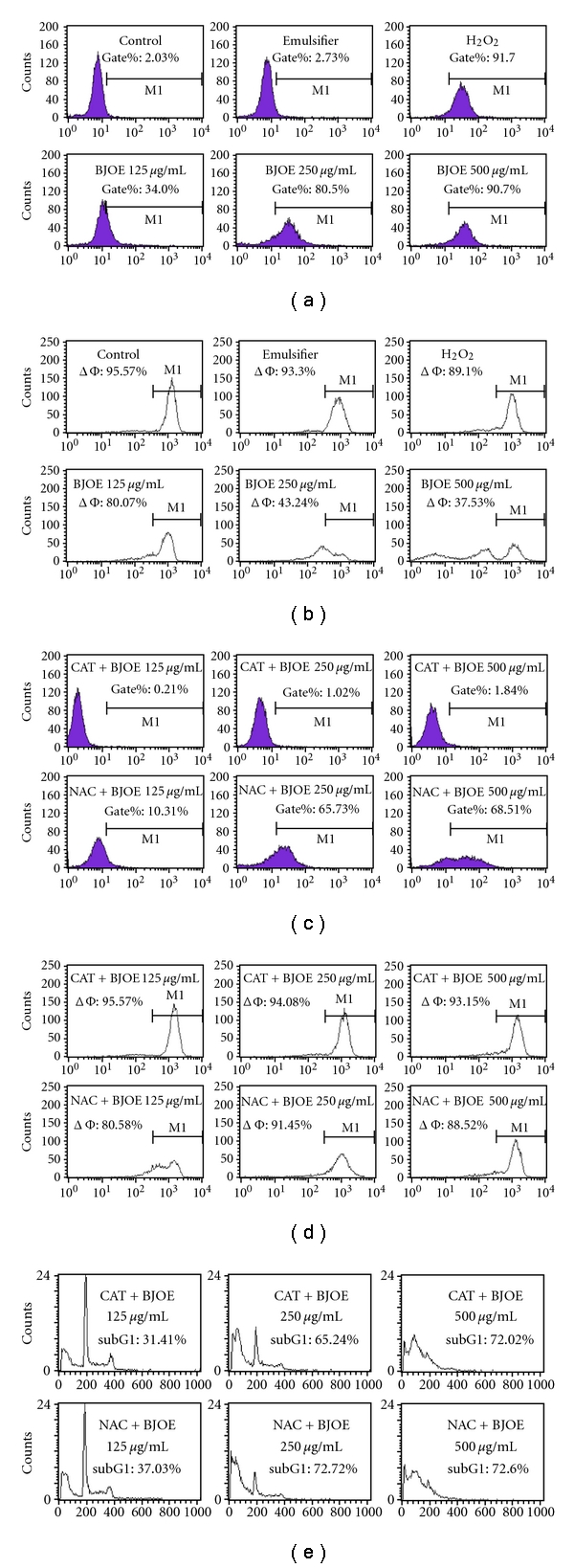
*Effect of BJO on ROS release and mitochondrial membrane potential (MMP) collapse in U937 cells.* U937 cells were treated with etoposide (10 *μ*mol/L) or various concentrations of BJO as indicated for 6 h. (a) U937 cells were labeled with 5 *μ*mol/L DCFH-DA for 1 h prior to the treatment with BJO. The addition of 100 *μ*mol/L H_2_O_2_ for 1 h was used as a positive control for the H_2_O_2_ level. (b) Disruption of MMP was determined according to changes in fluorescence density using rhodamine 123. (c, d, and e). ROS accumulation, MMP, and cell cycle examination of U937 pretreated with antioxidants. Cells were pretreated with catalase (500 U/mL) or N-acetylcysteine (10 mM) for 4 hours, followed by treatment with or without BJO for another 6 h. ROS level, MMP, and cell cycle were measured using flow cytometry as described in Materials and Methods.

ROS levels were determined in U937 and HL-60 cells after BJO treatment using a sensitive fluorescent probe, DCFH-DA. Intracellular ROS levels increased in a concentration-dependent manner after BJO treatment for 6 h (Figure 2(a)). Since the impairment of mitochondrial function has been considered to be a key event in the ROS-mediated apoptotic pathway [25, 26], the MMP in U937 and HL-60 was determined. As shown in Figure 2(b), a significant reduction in MMP was observed in U937 cells. Similar results were also observed in HL-60 cells (data not shown). These data suggest that BJO may induce apoptosis through a mitochondria-mediated pathway due to ROS production. 

To examine whether the apoptosis induced by BJO was uniquely caused by MMP collapse, two antioxidants, catalase (CAT) and N-acetylcysteine (NAC, a precursor of reduced glutathione and a free radical scavenge) were used. Neither NAC nor CAT *per se* regulated apoptosis, ROS production, or MMP in U937 and HL-60 cells, respectively, (data not shown). Pretreatment with either CAT or NAC effectively reversed the increase in ROS release and decrease in MMP in U937, which was induced by BJO treatment for 6 h (Figures 2(c) and 2(d)). However, neither CAT nor NAC affected the apoptosis induced by BJO (Figure 2(e)). Similar results were also observed in HL-60 cells (data not shown). These results suggested that the reduced MMP caused by ROS production may not be the only downstream effect for BJO-induced apoptosis. 

### 3.3. BJO-Induced Apoptosis in U937 Cells Accompanied by a Reduction in Protein Levels of c-FLIP_(L/S)_, Mcl-1, Bcl-2, XIAP, and Survivin

**Figure 3:**
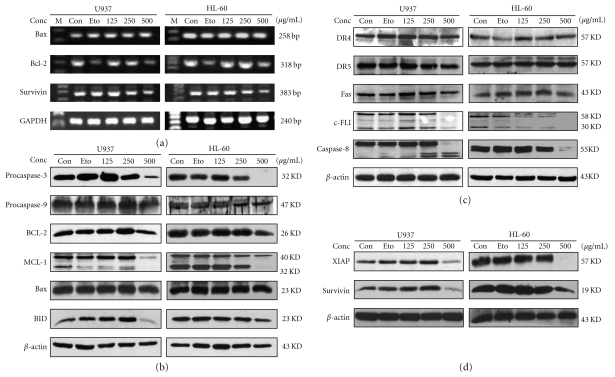
*Effect of BJO on apoptotic proteins.* AML cells were treated with etoposide (10 *μ*mol/L) and various concentrations of BJO as indicated for 6 hours. (a) The mRNA levels of apoptosis-related proteins were examined by RT-PCR as described in Materials and Methods. (b, c, and d) Western blotting analysis of procaspase-3, procaspase-9, Bcl-1, Mcl-1, Bax, Bid, pro-caspase-8, c-FLIP_(L/S)_, DR4, pro-DR5, Fas, XIAP and Survivin. Lane 1 and Lanes 3–5: cells were treated with or without BJO. Lane 2: cells were treated with etoposide (10 *μ*mol/L). The level of each protein was determined using specific antibodies as described in Materials and Method.

Caspases have been shown to play critical roles in the initiation and maintenance of apoptosis [27]. Thus, the activation of caspases in BJO-treated cells was examined. As shown in Figures 3(a) and 3(b), BJO activated procaspase-3 resulting in the cleavage of PARP. Moreover, as the caspase member acting on the mitochondrial pathway, the protein levels of procaspase-9 were reduced after BJO treatment. Caspase-8 was also activated by BJO (Figure 3(b)).

To discover the relationship between BJO and the activation of the death receptor pathway, the death receptors including Fas, DR4, and DR5 were examined. As shown in Figure 3(c), the expression of Fas, DR4, or DR5 was not altered by BJO. c-FLIP_(L/S), _which is an inhibitor of apoptosis downstream of the death receptors Fas, DR4, and DR5 [28], was significantly reduced after BJO treatment, which might cause the activation of caspase-8 (Figure 3(c)). It was also found that Bid, which is not only a substrate of caspase-8 but also a bridge protein of the mitochondrial pathway and death receptor pathway, was also cleaved by BJO (Figure 3(b)). These results suggest that the activation of the death receptor pathway by BJO may be caused by the downregulation of c-FLIP_(L/S)_. 

To examine the relationship between BJO and mitochondrial disruption, the Bcl-2 family antiapoptotic proteins which are the critical determinants of mitochondrial-dependent caspase activation were examined [29]. As shown in Figures 3(a) and 3(b), the mRNA level of Bcl-2 and the protein levels of Bcl-2 and Mcl-1 were reduced following BJO treatment, while the protein level of Bax remained unchanged. This suggests that the activation of the mitochondrial pathway may be caused by the downregulation of Mcl-1 and Bcl-2. 

In addition, BJO significantly reduced the protein levels of XIAP and survivin (Figures 3(a) and 3(d)). Both of them are inhibitors of caspase-9 and caspase-3 [30]. The mRNA levels of survivin were also reduced (Figure 3(a)). Taken together, these data indicated that BJO-induced apoptosis in U937 and HL-60 cells activated both the mitochondrial and the death receptor pathway, accompanied by downregulation of c-FLIP_(L/S)_, Mcl-1, Bcl-2, and activation of Bid. Also, the reduction in XIAP and survivin could enhance the activity of the caspases. 

### 3.4. BJO Introduces Apoptotic Death of Primary Human Leukemic Cells

**Figure 4:**
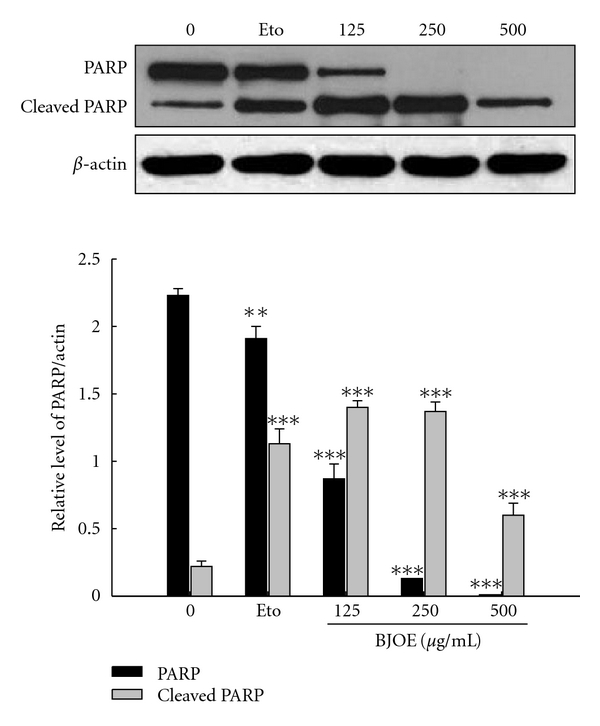
Western blotting analysis of PARP in primary leukemia cells from AML patients. The cells were treated with or without BJO. *β*-actin was also detected as the loading control. Data were represented as mean ± SE of three separate experiments, ***P < .001. The data was a representative of one result of fifteen patients.

Since BJO treatment can induce apoptosis in AML cell lines, it would be interesting to see whether BJO could be used clinically to treat AML. The apoptotic effect of BJO on AML patients' leukemic cells (including M2 and M5) was examined *in vitro* using FACS and Western blotting analysis. All 15 samples were treated with BJO for 6 h. As Table 1 shows, the percentages of cells in the subG1 phase were increased in a concentration-dependent manner in all samples. In addition, as shown in Figure 4, cleaved PARP can also be seen in 7 of the 15 cases. These results indicated that BJO also induced apoptosis in human primary leukemic cells. 

### 3.5. BJO Is Relatively Noncytotoxic to Normal Peripheral Blood Lymphocytes

**Figure 5:**
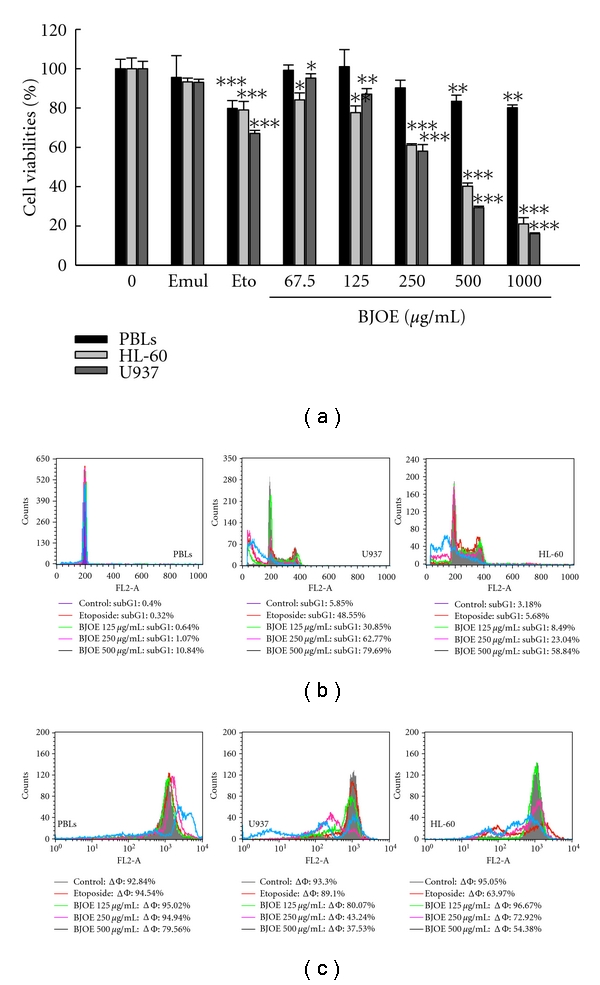
*Effect of BJO on human peripheral blood lymphocytes and AML cells.* AML cells and PBLs were treated with emulsifier (negative control), etoposide (10 *μ*mol/L, positive control), or indicated concentrations of BJO for 6 hours. (a) Cell viabilities were determined by MTT assay. ***P < .001 (b) Apoptotic cells were evaluated by cell cycle analysis. Percentage of cells in the subG1 phase was measured using FACS as described in Materials and Method. (c) Disruptions of MMP were determined by changes in fluorescence density using rhodamine 123.

To further examine the cytotoxic effect of BJO on normal cells, we compared the cytotoxicity of BJOE on AML cells and normal PBLs using MTT. As shown in Figure 5(a), the cell viabilities of U937 and HL-60 were much lower than that of PBLs after BJO treatment, suggesting that the AML cells were more sensitive to BJO. The selectivity index (SI) of BJO, which was determined from the ratio of the IC_50_ of the PBLs cells to that of the AML cells [31], was more than 4.0. In addition, the cell numbers of PBLs in the subG1 phase were much lower than those of AML cells (Figure 5(b)). Since the mitochondrial pathway is involved in chemotherapy-induced apoptosis [32], we also found that the percentages of cells in PBLs with low MMP were much lower than those in AML cells (Figure 5(c)). These results suggested that BJO selectively induces apoptosis in AML cells with little toxicity to normal haemopoietic cells. 

### 3.6. Antileukemia Effect of BJO against U937 Leukemia Xenograft in Nude Mice

**Figure 6:**
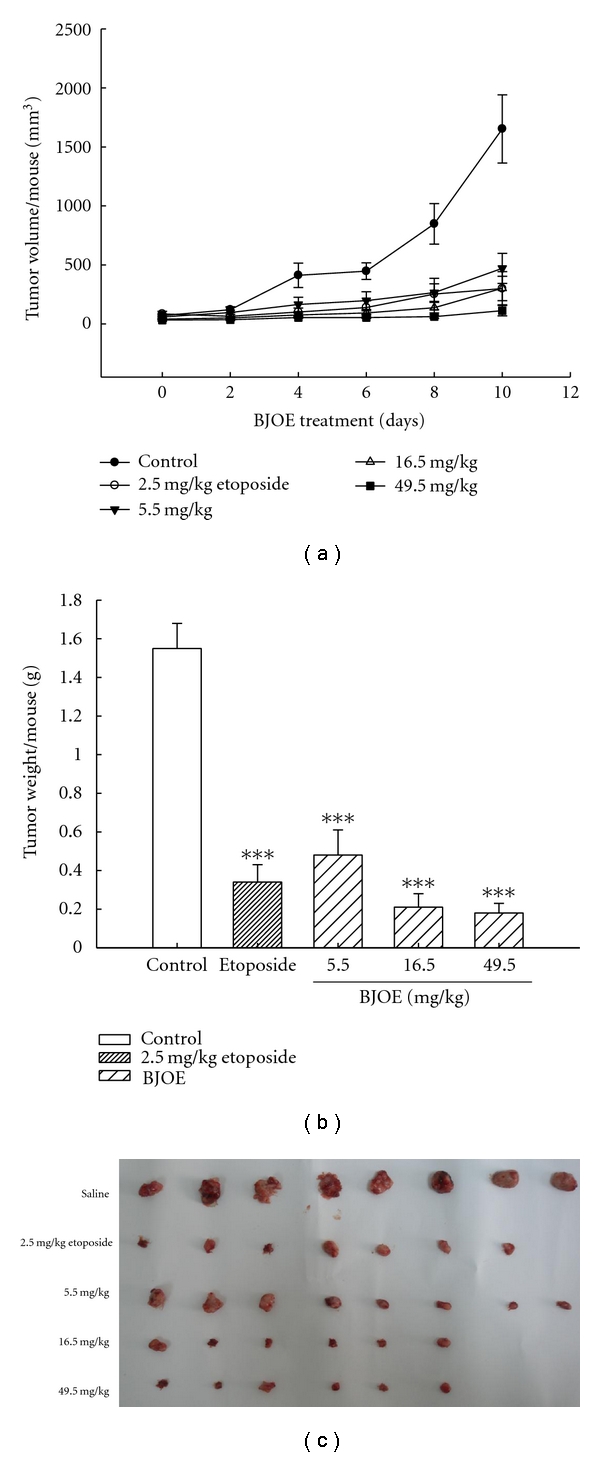
*BJO inhibits the growth of mouse tumors in U937 xenograft model.* (a) U937 cells (3 × 10^7^/200 *μ*L PBS per mouse) were injected s.c. into the right flank of mice. Mice were injected with saline or designated doses of BJO as described in Materials and Methods. Tumor size was measured once every two days with a caliper (calculated volume = shortest diameter^2^× longest diameter/2). (b) 11th day after intravenously injection BJO, mice were sacrificed and the tumors were excised and weighed. ***P < .001. (c) The picture of tumor in nude mice with or without treatment of BJO.

We further evaluated the antileukemia ability of BJO in a mouse U937 tumor model. U937 cells were subcutaneously injected into the right flank of Balb/c nude mice and rapidly gave rise to exponentially growing tumors. When the average tumor volume reached 100 mm^3^, the nude mice were intravenously injected with saline, etoposide (2.5 mg/kg), or BJO (5.5, 16.5 and 49.5 mg/kg). After 10 days, the mean volume of tumors in mice treated with BJO was much smaller than the tumors in the saline-treated mice (Figure 6(a)). Correspondingly, tumor weight in BJO-treated groups was also significantly smaller compared with the saline-treated mice (Figure 6(b)).The tumor in nude mice with or without BJO treatment is shown in Figure 6(c). These results suggested that BJO also has a significant antileukemia effect *in vivo*.

### 3.7. The Active Components of *Brucea javanica* Oil

**Figure 7:**
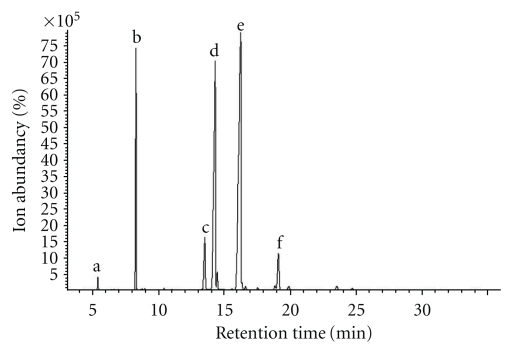
*Total contents of Brucea Javanica oil emulsion.* The content of BJOE was analyzed by gas chromatography-mass spectrometry as described in Materials and Methods. The percentages of six components in BJOE were phenol (a; C_6_H_6_O, 0.59%), hexadecanoic acid (b; C_17_H_34_O_2_, 11.75%), octadecanoic acid (c; C_19_H_38_O_2_, 5.45%), 9-octadecenoic acid (d; C_19_H_36_O_2_, 29.24%), 9,12-octadecadienoic acid (e; C_19_H_34_O_2_, 44.85%), and 9,12,15-octadecatrienoic acid (f; C_19_H_32_O_2_, 4.22%). Representative graph of two runs.

**Figure 8:**
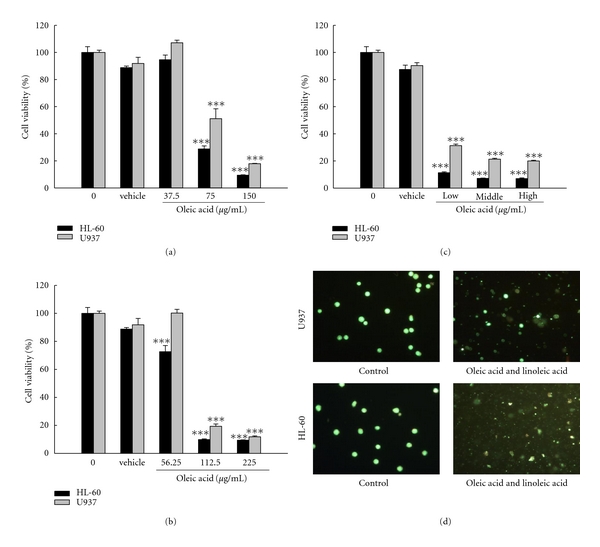
*Active contents of Brucea Javanica oil.* (a) HL-60 and U937 cells were treated with oleic acid (37.5, 75, and 150 *μ*g/mL) as indicated for 6 h. (b) HL-60 and U937 cells were treated with linoleic acid (56.25, 112.5, 225 *μ*g/mL) as indicated for 6 h. (c) HL-60 and U937 cells were treated with the mixture of oleic acid and linoleic acid as the the same ratio as in BJO (low:oleic acid 37.5 *μ*g/mL & linoleic acid 56.25 *μ*g/mL; Middle: oleic acid 75 *μ*g/mL & linoleic acid 112.5 *μ*g/mL; High: oleic acid 150 *μ*g/mL & linoleic acid 225 *μ*g/mL) for 6 h. (d) U937 and HL-60 cells morphology analysis following treatment with the mixture of oleic acid and linoleic acid. AO/EB was used to stain cells and the cellular fluorescent changes were observed using fluorescence microscope. Results were obtained from three separate experiments.

The total components of BJOE were measured by GC-MS. As shown in Figure 7, there were six components in BJOE: phenol (0.59%), hexadecanoic acid (11.75%), octadecanoic acid (5.45%), 9-Octadecenoic acid (oleic acid) (29.24%), 9E,12Z-Octadecadienoic acid (linoleic acid) (44.85%), and 9Z,12Z,15Z-Octadecatrienoic acid (4.22%) (Table 2). Among the six components, oleic acid and linoleic acid were two major components. As shown in Figures 8(a) and 8(b), both oleic acid and linoleic acid had cytotoxic effect on HL-60 and U937 cells. Moreover, the mixture of oleic acid and linoleic acid at the same ratio as in BJO also has cytotoxic effect (Figure 8(c)). The cells treated with the mixture at different concentrations also demonstrated morphology characteristic of apoptosis, such as chromatin condensation and formation of apoptotic bodies (Figure 8(d)). Since BJOE was composed of BJO and soybean phospholipids, and the emulsifier soybean phospholipids do not have cytotoxic effect, these results suggested that the active components of BJO were oleic acid and linoleic acid.

## 4. Discussion

Naturally occurring drugs are playing a significant role in treating AML, including homoharringtonine and etoposide [14]. While clinical reports had shown that both of them can induce many adverse effects resulting in the decrease of the life quality and aggravate the syndrome, like myelosuprression. The emulsion of BJO had been clinically used to treat carcinoma for many years, and the clinical reports also showed that combination of the emulsion of BJO with chemotherapy can not only increase the anticancer effect but also decrease the toxicity of chemotherapy [15, 16].

Although the emulsion formulation of BJO has been used clinically in China for more than 20 years to treat carcinoma, the active components and the molecular mechanisms remained unknown. The present study first showed that BJO produced a concentration-dependent cytotoxicity to AML cell lines U937 and HL-60. Cell cycle examination, cleaved PARP, and DNA fragmentation analysis indicated that the cytotoxic effect of BJO was mediated by induction of apoptosis. 

**Figure 9:**
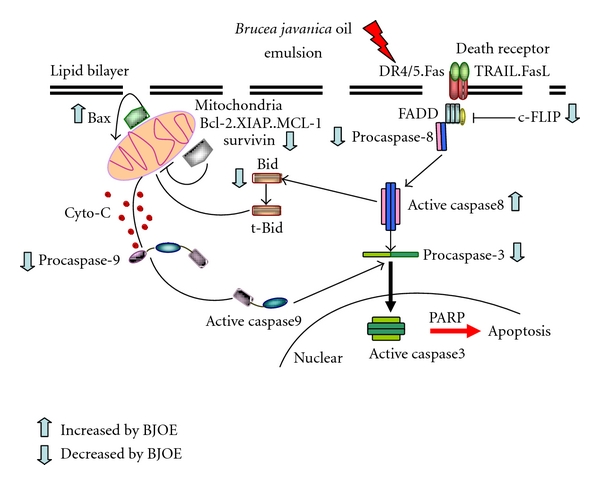
A schematic illustration of signal pathways involved BJO-induced cell apoptosis.

The multiple pathways involved in the action of BJO were further identified (Figure 9). Firstly, BJO induced mitochondria-initiated apoptosis, including reduction in MMP and ROS production. Secondly, BJO treatment downregulated c-FLIP_(L/S)_, an inhibitor of the death receptors Fas, DR4, and DR5 [28], and subsequently led to the activation of caspase-8. Thirdly, BJO also downregulated XIAP and survivin, two important members of the IAPs family [30, 33], and subsequently led to the activation of caspase-9 and caspase-3. Finally, BJO decreased mRNA and protein expressions of Mcl-1 and Bcl-2, two members of the antiapoptotic Bcl-2 family proteins. Since the Bcl-2 family proteins are critical determinants of mitochondrial-dependent caspase activation [29], downregulation of Mcl-1 and Bcl-2 may also contribute to mitochondria-initiated apoptosis. Taken together, it could be proposed that BJO induced apoptosis in U937 and HL-60 cells via activation of the death receptor pathway by downregulation of c-FLIP_(L/S)_, and activation of the mitochondrial pathway by downregulation of Mcl-1 and Bcl-2. In addition, the reduction of XIAP and survivin could enhance the activity of the caspases (Figure 9). All these findings strongly suggest that BJO can be a potential chemotherapeutic drug for AML. 

In addition to the therapeutic effect of anticancer drugs on malignant cells, chemotherapy usually causes severe toxicity to normal tissues. The present study showed that the cytotoxic activity of BJO on normal PBLs was much weaker than that on AML cells. After BJO treated, the cell numbers of PBLs in the subG1 phase were much lower than those of AML cells. Similarly, BJO treatment induced MMP collapse in AML cells as well as in normal PBLs, but to a much lesser extent. This suggests that BJO exhibits more selective cytotoxicity to AML cells [34]. 

According to the FAB classification, the monocytic leukemia cell line U937 belongs to the AML subtype of M5 and HL-60 belongs to M2 [31]. By using primary leukemic cells from M5 and M2 patients, we found that BJO could increase the percentage of subG1 cells, and 7 of 14 M5 patients' leukemia cells were sensitive to BJO *in vitro* (>50% apoptosis). The cleavage of PARP induced by BJO treatment also confirmed that BJO could induce apoptosis in primary leukemic cells from AML patients. Both the selective cytotoxicity and apoptosis inducting effect of BJO imply the therapeutic application of BJO in the treatment of AML patients.

Moreover, we further examined the *in vivo* anticancer effect of BJO in a nude mouse tumor model. BJO effectively slowed the tumor growth, even at a lower dose. In the BJO-treated group, both tumor volume and weight were significantly reduced compared with that of the control group. These results further confirmed the anticancer effect of BJO and its clinical potentials. 

The total BJO components were determined by gas chromatography-mass spectrometry (GC-MS) and it was found that oleic acid and linoleic acid were two major components. It has been reported that oleic acid and linoleic acid can induce apoptotic death of breast cancer, lung cancer prostate cancer, and lymphoma cells [35–38]. In our study, we found that both oleic acid and linoleic acid has cytotoxic effect on AML cell lines HL-60 and U937 cells. Thus we propose that the antileukemic effect of BJO result mainly from the existence of oleic acid and linoleic acid. 

In summary, the present study first demonstrated that BJO possessed AntiLeukemia effect both in vitro and in vivo. BJO induced AML cell apoptosis via both the mitochondrial and death receptor apoptosis pathway by downregulating c-FLIP_(L/S)_, Mcl-1, Bcl-2, surviving, and XIAP. The data also showed that oleic acid and linoleic acid were the active components of BJO. These combined observations suggest that BJO has a high potential for clinical application in the treatment of AML.

## Figures and Tables

**Table 1:** Clinical data of AML patients and apoptosis rate induced* in vitro* by BJO.

Patient no.	Sex/Age	HB (g/L)	WBC (×10^9^)	Platelet (×10^12^/L)	APL Cell (%) in BM	AML type	Apoptosis rate (%) (500 *μ*g/mL)
1	F/22	44	57.8	1.23	67	M5	51.31
2	M/58	109	30.0	1.73	77.5	M5	38.98
3	M/65	98	283.3	3.80	86	M5	53.79
4	F/25	64	125.0	18.6	54	M5	38.04
5	F/55	116	255	3.20	91.5	M5	96.23
6	M/65	66	171.6	1.9	67	M5	45.09
7	M/50	128	247	3.3	68.5	M5	53.44
8	M/46	84	26.3	2.3	88.5	M5	75.92
9	M/45	46	1.7	1.23	50	M5	28.80
10	M/63	50	75.0	1.28	76.8	M5	90.04
11	M/63	64	42.9	1.8	52	M5	53.79
12	F/47	85	439.6	3.1	52	CML-M5	29.35
13	M/59	77	23.3	2.67	27.5	CML-M5	92.48
14	F/45	56	41.9	3.45	26.4	CML-M5	63.84
15	F/62	96	235.6	3.8	90.0	M2	64.26

Note: The patients' leukemic cells were treated with or without BJO for 6 h. Percentage of cells in the subG1 phase was measured using FACS as described in Materials and Method.

**Table 2:** The component of BJOE.

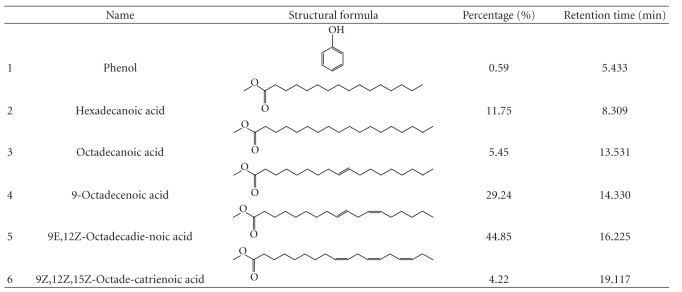

Note: The total components of BJOE were measured by GC-MS as described in Materials and Method.
